# Remifentanil use in critically Ill patients requiring mechanical ventilation is associated with increased delirium-free days: a retrospective study

**DOI:** 10.1186/s12245-025-00846-y

**Published:** 2025-03-19

**Authors:** Junpei Haruna, Aki Sasaki, Satoshi Kazuma

**Affiliations:** 1 Department of Intensive Care Medicine, School of Medicine, Sapporo Medical University, S-1, W-16, Chuo-Ku, Sapporo, Hokkaido 060-8543 Japan; 2 Department of Nursing, Sapporo Medical University Hospital, Sapporo, Hokkaido 060-8543 Japan

**Keywords:** Remifentanil, Delirium, Critical illness

## Abstract

Intraoperative remifentanil reduces postoperative delirium incidence, unlike other opioids; however, its efficacy in medical emergencies with organ failure is unknown. We hypothesized that remifentanil use in nonoperative intensive care unit (ICU) patients requiring ventilatory management would improve delirium outcomes. This retrospective study included 95 nonoperative patients with unplanned ICU admissions requiring ventilatory opioids. Delirium was assessed using the Intensive Care Delirium Screening Checklist. Patients were divided into remifentanil and non-remifentanil groups; statistical adjustments were made using propensity score matching and inverse probability weighting. After matching, the remifentanil group had significantly more delirium-free days (DFDs) within 14 days than the non-remifentanil group (8 [5–11] vs. 5 [3–9], *p* < .001). Adjusted multivariate analysis showed that DFD was significantly increased in the remifentanil group (Odds ratio = 2.639 [95% CI 1.279–5.445]; *p* = 0.009). Remifentanil use in nonoperative ventilated ICU patients may reduce delirium duration.

**Supplementary Information:**

The online version contains supplementary material available at 10.1186/s12245-025-00846-y.

## Introduction

Delirium in critically ill patients increases morbidity, hospital stays, and costs. Analgesic choice during ventilation may affect delirium incidence and duration. Remifentanil, an ultra-short-acting opioid, has been reported to reduce the incidence of postoperative delirium when used intraoperatively compared to other opioids [1]. Its rapid metabolism and short context-sensitive half-life result in less accumulation and quicker recovery, potentially minimizing delirium risk.

In the intensive care unit (ICU) setting, particularly among patients requiring mechanical ventilation, the benefits of remifentanil have been explored. Some studies have suggested that remifentanil minimizes the need for sedatives like midazolam and dexmedetomidine during mechanical ventilation, thereby potentially reducing delirium incidence [2]. However, randomized controlled trials have not consistently demonstrated remifentanil's superiority over fentanyl in analgesia protocols or in reducing delirium [3]. Meta-analyses have also yielded inconclusive results regarding remifentanil's advantages in this context [4].

Remifentanil's shorter half-life compared to fentanyl and morphine may allow for a more predictable and shorter duration of mechanical ventilation in critically ill patients [4, 5]. In surgical ICU patients expected to be extubated sooner, the benefits of remifentanil might be less pronounced due to the necessity for post-extubation analgesia using longer-acting opioids. Therefore, remifentanil's pharmacological effects may be more fully realized in medical ICU patients requiring prolonged ventilation, such as those with acute respiratory or circulatory failure.

Moreover, remifentanil use in the ICU may improve clinical outcomes related to delirium by reducing sedative sdose and providing effective analgesia without accumulation. Despite this, most previous ICU studies have focused on perioperative patients, including scheduled postoperative patients [1, 2, 4, 6], and data on nonoperative patients are limited. The efficacy of remifentanil in medical emergencies involving organ failure remains unclear. Therefore, we hypothesized that remifentanil use in nonoperative patients requiring mechanical ventilation in the ICU would result in favorable delirium outcomes.

## Methods

### Study design and setting

This single-center, retrospective observational study was conducted at a university-affiliated tertiary care hospital with a 20-bed ICU. The study period spanned from September 2022 to December 2023. Patient confidentiality was maintained, and data were anonymized. This study was conducted and reported in accordance with the STROBE guidelines (Appendix 1). The Sapporo Medical University Hospital Institutional Review Board has approved the research protocol (approval number 362–81, 2024), and the need for informed consent was waived due to the retrospective nature of the study.

### Patient selection

We included adult patients (≥ 18 years) with unplanned ICU admissions for non-surgical reasons who required invasive mechanical ventilation (i.e. intubation rather than non-invasive mechanical ventilation [e.g. CPAP])and received opioid analgesia with either remifentanil or fentanyl. Exclusion criteria were scheduled ICU admissions, patients not receiving mechanical ventilation, those not administered fentanyl or remifentanil, and patients with incomplete medical records. The choice between remifentanil and fentanyl was at the discretion of the attending physician.

### Data collection

Two trained investigators independently reviewed all medical records. Any disagreements were resolved through discussion with a third investigator. We reviewed electronic medical records to collect baseline demographic data, including age, sex, height, weight, and BMI. Clinical data at ICU admission included the reason for admission, ICU length of stay (LOS), Daily mean RASS scores, Acute Physiology and Chronic Health Evaluation II (APACHE II) score, and Sequential Organ Failure Assessment (SOFA) score. Treatments such as continuous renal replacement therapy (CRRT) were recorded.

Medication data included dosage parameters for remifentanil, fentanyl, noradrenaline, midazolam, propofol, and dexmedetomidine. Sedation levels were assessed using the Richmond Agitation–Sedation Scale (RASS), and analgesia was evaluated with the Critical Care Pain Observation Tool (CPOT), documented every four hours. We recorded days of deep sedation (RASS -4 to -5), days of agitation (RASS 1 to 4), and maximum CPOT scores during ventilation.

### Outcome measures

The primary outcomes were the occurrence of delirium, defined as an Intensive Care Delirium Screening Checklist (ICDSC) score of ≥ 4, and the number of delirium-free days (DFDs) within 14 days from ICU admission. The secondary outcome was the number of ventilator-free days (VFDs) within 14 days.

### Statistical analysis

Patients were categorized into remifentanil and non-remifentanil groups based on the analgesic regimen during mechanical ventilation. Continuous variables are presented as medians with interquartile ranges (IQRs), and categorical variables as counts and percentages. Comparisons between groups were made using the Mann–Whitney U test for continuous variables and the chi-squared or Fisher's exact test for categorical variables.

To minimize potential confounders and selection bias, we performed propensity score (PS) matching using logistic regression. Variables included were age, sex, BMI, cognitive dysfunction, APACHE II score, SOFA score, ICU LOS, presence of sepsis, use of CRRT, and administration of dexmedetomidine, midazolam, and propofol. A 1:1 nearest-neighbor matching without replacement and a caliper width of 0.2 of the standard deviation of the logit of the PS were used. Balance was assessed using standardized mean differences (SMDs), with values less than 0.10 indicating adequate balance.

We also applied inverse probability of treatment weighting (IPTW) to adjust for confounders. Multivariate logistic regression analyses were conducted before and after IPTW adjustment to evaluate the association between remifentanil use and outcomes, including the same covariates used in PS matching.

A post-hoc power analysis determined that the sample size of 95 patients provided a power of 0.929 to detect a significant difference in DFDs, exceeding the required 73 patients. Statistical analyses were performed using SPSS Statistics version 27 (IBM Corp., Armonk, NY, USA), with a two-tailed *p*-value of < 0.05 considered statistically significant.

## Results

### Patient characteristics

Out of 857 ICU admissions, 95 patients met the inclusion criteria. Thirty-two patients (33.7%) received remifentanil, while 63 received fentanyl (Appendix 2). Before PS matching, the remifentanil group had a longer ICU LOS (median 8.5 days [IQR 6–11] vs. 6.5 days [IQR 4–9]; *p* = 0.041) and higher dexmedetomidine use (93.8% vs. 71.4%; p = 0.031) compared to the non-remifentanil group (Table 1).

**Table 1:** Basic characteristics and outcomes of patients before and after matching

		Before Matching (*n* = 95)				After Matching (*n* = 62)			
	Overall (*n* = 95)	Remifentanil Group (*n* = 32)	Non remifentanil Group (*n* = 63)	*P*-value	SMD	Remifentanil Group (*n* = 31)	Non remifentanil Group (*n* = 31)	*P*-value	SMD
Age (years), median [IQR]	71.5 [59.5–77.3]	70.5 [57–77]	72 [61.3–79.5]	0.085	- -0.13	71 [76–77]	73 [61–81]	0.451	0.10
Male, n (%)	52 (54.7)	17 (53.1)	33 (52.4)	0.831	-0.04	17 (53.1)	15 (48.4)	0.800	0.09
BMI, median [IQR]	23.1 [20.4–27.1]	22.5 [20.6–26.7]	23.2 [19.9–27.2]	0.093	-0.02	22.7 [20.5–27.1]	22.3 [19.6–25.3]	0.464	-0.09
Reasons for ICU admission									
Respiratory failure, n (%)	34 (35.8)	15 (46.9)	19 (14.3)	0.119	0.35	14 (45.1)	13 (41.9)	1.000	0.07
Sepsis, n (%)	22 (23.2)	8 (25.0)	14 (22.2)	0.800	0.07	7 (22.6)	7 (22.6)	1.000	0.07
Circulatory failure, n (%)	12 (12.6)	4 (12.5)	8 (12.7)	0.800	-0.01	4 (12.9)	4 (12.9)	1.000	0.05
Cerebrovascular disease, n (%)	15 (15.8)	3 (9.3)	12 (19.0)	0.150	-0.37	3 (9.3)	5 (16.1)	0.707	-0.27
Acute pancreatitis, n (%)	5 (5.3)	4 (12.5)	1 (1.6)	0.043	0.49	4 (12.5)	1 (3.2)	0.354	0.5
Acute kidney injury, n (%)	3 (3.2)	2 (6.3)	1 (1.6)	0.262	0.27	2 (6.4)	1 (3.2)	1.000	0.15
Endocrine disease, n (%)	2 (2.1)	1 (3.1)	1(1.6)	1.000	0.11	1 (3.2)	0(0.0)	0.492	0.25
Metabolic disorder, n (%)	2 (2.1)	1 (3.1)	1 (1.6)	1.000	0.11	1 (3.2)	0 (0.0)	0.492	0.01
Past medical history									
Alcohol dependence, n (%)	1 (0.01)	0 (0)	1 (0.02)	1.000	-	0 (0)	0 (0)	1.000	-
Cognitive dysfunction, n (%)	7 (7.4)	0 (0)	7 (11.1)	0.09	-	0 (0)	0 (0)	1.000	-
APACHE II at ICU admission, median [IQR]	17 [13–22]	17 [13–24]	17 [13–22]	0.309	0.27	18 [14–25]	17 [5–10]	0.938	0.07
SOFA at ICU admission, median [IQR]	7 [5–9]	7 [5–10]	7 [4–9]	0.944	0.16	7 [5–10]	7 [4–12]	0.972	0.06
ICU length of stay (days), median [IQR]	6 [4–10]	8.5 [6–11.8]	6.5 [3–12]	0.041	0.58	8.5 [6–12]	7 [4–12]	0.203	0.15
ICU mortality, n (%)	8 (8.4)	3 (9.4)	5 (7.9)	0.831	0.05	3 (9.7)	2 (6.5)	1.000	0.1
28-day mortality, n (%)	19 (20.0)	5 (15.6)	14 (22.2)	0.590	-0.16	5 (15.6)	7 (22.6)	0.749	-0.1
Prone position, n (%)	7 (7.4)	5 (15.6)	2 (3.2)	0.041	0.48	5 (16.1)	1 (3.2)	0.195	0.31
Tracheotomy, n (%)	22 (23.2)	6 (18.8)	5 (20.0)	0.609	0.34	6 (19.4)	7 (22.6)	1.000	0.12
CRRT, n (%)	27 (28.4)	12 (37.5)	15 (23.8)	0.228	0.30	11 (35.5)	8 (25.8)	0.416	0.10
Drug use and dose									
Remifentanil									
Use of remifentanil, n (%)	32 (33.7)	-	-	-	-	31 (100)	-	-	-
Infusion total dose (ug)	965 [491–2652]	-	-	-	-	981[474–2700]	-	-	-
Dosing duration (h)	73 [47–149]	-	-	-	-	78 [49–158]	-	-	-
Infusion rate-mean (ug/kg/min)	0.029 [0.02–0.06]	-	-	-	-	0.029 [0.02–0.06]	-	-	-
Fentanyl									
Use of fentanyl, n (%)	88 (92.6)	20 (62.5)	63 (100.0)	< .001	-	20 (64.5)	31 (100)	< .001	-
Infusion total dose (ug)	829 [533–2235]	766 [101.7–947.9]	1523 [520–2780]	< .001	-	765 [101.9–992.1]	1678 [926–3620]	< .001	-
Dosing duration (h)	90 [41–163]	90 [42–209]	92 [45–178]	0.698	-	90 [42–207]	100 [48–192]	0.188	
Infusion rate-mean (ug/kg/h)	0.16[0.14–0.20]	0.15 [0.14–0.19]	0.17 [0.14–0.20]	0.265	-	0.15 [0.14–0.19)	0.17 [0.15–0.19]	0.597	
Use of noradrenaline, n (%)	56 (58.9)	21 (65.6)	34 (54.0)	0.379	-	21 (67.7)	20 (64.5)	0.786	-
Use of dexmedetomidine, n (%)	75 (78.9)	30 (93.8)	45 (71.4)	0.031	0.55	29 (93.5)	28 (90.3)	1.000	0.10
Use of midazolam, n (%)	35 (36.8)	14 (48.3)	21 (33.3)	0.371	0.15	13 (41.9)	12 (38.7)	1.000	0.07
Use of propofol, n (%)	68 (72.3)	23 (74.2)	45 (71.4)	1.000	0.01	22 (73.3)	24 (77.4)	0.772	-0.09
Sedation and analgesic status									
Number of days for RASS-4 and -5	0 [0–3]	0 [0–6]	2 [0–7.5]	< .001	-	0 [0–7]	0 [0–8]	0.121	-
Dairy mean RASS Score	-1 [0—-2]	-1 [0- -2]	-2 [0—-2]	0.42	-	-1 [0- -2]	-2 [0—-2]	0.489	-
Number of days for RASS 0 to 4	0 [0–0]	0 [0–0]	2 [0–2]	0.187	-	0 [0–0]	0 [0–0]	0.687	-
Maximum CPOT, median [IQR]	1 [0–2]	0 [0–1]	1 [0–2]	0.848	-	0 [0–2]	0 [0–2]	0.832	-
Inflammation response data									
IL-6 at ICU admission (pg/ml)	226 [80.6–1252.5]	258 [105–1723]	175 [58.6–1195]	0.335	-	256 [104–1990]	291 [30.5–753.8]	0.404	-
CRP at ICU admission (mg/dL)	8.8 [2–17.5]	12.1 [7.7–22.2]	7.0 [1.7–15.3]	0.01	-	12.1 [7.7–22.2]	6.8 [2.0–17.6]	0.061	-
CRP at ICU days 3 (mg/dL)	11.7 [4.9–17]	12.1 [6.5–18.9]	11.2 [4.1–16.8]	0.292	-	11.7 [6.0–17.0]	12.6 [3.2–16.1]	0.508	-
CRP at ICU discharge (mg/dL)	7.1 [2.5–11.6]	4.6 [1.8–11.5]	7.2 [3.7–12]	0.122	-	4.5 [1.8–11.1]	8.8 [4.8–15.9]	0.039	-

*Abbreviations*: *APACHE II* Acute Physiology and Chronic Health Evaluation II, *CPOT* Critical-Care Pain Observation Tool, *CRP* C-reactive protein, *CRRT* continuous renal replacement therapy, *IL-6* Interleukin-6, *IQR* Interquartile range, *SMD* Standardized Mean Difference, *RASS* Richmond Agitation- Sedation Scale, *SOFA* sequential organ failure assessment

### Propensity score matching

PS matching resulted in 31 matched pairs, achieving adequate covariate balance (SMD < 0.10 for all variables). The c-statistic for the PS model was 0.80, indicating good discrimination.

### Outcomes

After matching, no patients in either group received morphine; however, 20 patients in the remifentanil group also received fentanyl. The remifentanil group had significantly more DFDs within 14 days compared to the non-remifentanil group (median 8 days [IQR 5–11] vs. 5 days [IQR 3–9]; *p* < 0.001) (Table 2). There was no significant difference in delirium occurrence between the groups (34.4% vs. 48.8%; *p* = 0.44).

**Table 2:** Outcome of patients before and after propensity score matching

-		Before Matching (*n* = 95)			After Matching (*n* = 62)		
	Overall (*n* = 95)	Remifentanil Group (*n* = 32)	Non remifentanil Group (*n* = 63)	*P*-value	Remifentanil Group (*n* = 31)	Non remifentanil Group (*n* = 31)	*P*-value
Ventilator free days at 14 days, median [IQR]	1 [0–2]	8 [4–11]	9 [7–12]	0.035	8 [4–11]	9 [4–11]	0.392
Delirium, n (%)	34 (36.2)	11 (34.4)	23 (37.1)	0.825	11 (34.4)	15 (48.4)	0.44
Delirium Free Days at 14 days, median [IQR]	6 [3–9]	8 [4.5–10.7]	6 [3–11]	0.011	8 [5–11]	5 [3–9]	< .001

*Abbreviations*: *APACHEII* Acute Physiology and Chronic Health Evaluation II, *CPOT* Critical-Care Pain Observation Tool, *IQR* Interquartile range, *RASS* Richmond Agitation-Sedation Scale, *SMD* Standardized Mean Difference, *SOFA* sequential organ failure assessment

Using IPTW-adjusted multivariate logistic regression, remifentanil use was associated with a significant increase in DFDs both before and after weighting (β = 0.986 [95% CI 0.043–1.928]; *p* = 0.041 before IPTW; Odds ratio = 2.639 [95% CI 1.279–5.445]; *p* = 0.009 after IPTW). No significant differences were observed in delirium occurrence or VFDs within 14 days.

## Discussion

This study investigated remifentanil's impact on delirium in nonoperative ICU patients on ventilation. Results showed remifentanil reduced delirium duration compared to fentanyl. In general, intraoperative use of remifentanil decreases the incidence of postoperative delirium [5]. Since the effects of remifentanil during anesthesia include post-awakening sedation and decreased incidence of delirium, this study suggests that remifentanil may also be effective in reducing agitation, a peripheral symptom of delirium. In support, a postoperative study demonstrated that treatment of agitation at emergence with remifentanil infusion is more effective than treatment with propofol, with shorter time to extubation and discharge from the post-anesthesia care unit [6]. Nonetheless, how remifentanil’s impact on suppressing the onset of agitation affects delirium remains to be studied. Remifentanil may possess anti-inflammatory properties and decrease inflammatory cytokines, which may help suppress delirium [7, 8].

A strength of this study is its focus on critically ill ICU patients. Most studies of analgesic management during mechanical ventilation with remifentanil have been limited to postoperative cardiovascular surgery [9]or postoperative abdominal surgery [10], with few focusing on critically ill patients, such as those with sepsis. Another strength of this study is that it focused on the duration of delirium in critically ill ICU patients subsequent to remifentanil administration. Considering delirium is easily encountered in daily practice and adverse events due to delirium are frequently reported, the use of remifentanil during ventilatory management of critically ill patients could be suggested as a strategy to reduce the duration of delirium.

Limitations include the retrospective, single-center design, which may limit generalizability. While our post-hoc power analysis suggested adequate power, the sample size may have been insufficient to fully account for all potential confounding factors related to delirium in critically ill patients (e.g., fever, oxygenation status, electrolyte imbalances, and fluid balance. Furthermore, the PS matching process reduced the effective sample size to 62, which was lower than the 73 initially determined by the power calculation. This reduction in sample size may have affected the robustness of the results. Future studies should consider this limitation by increasing the initial sample size to ensure sufficient power after matching. Additionally, manual chart review may have introduced data extraction errors and misclassification, which are inherent limitations of retrospective studies. Future research should implement double-review processes or automated data extraction to enhance accuracy.

The longer ICU LOS in the remifentanil group, despite increased DFDs, may be influenced by factors like illness severity not fully captured. Patients receiving remifentanil might have had more complex conditions necessitating prolonged ICU care but benefited from reduced delirium duration.

## Conclusion

In conclusion, the use of remifentanil in nonoperative critically ill patients receiving ventilation in the ICU may reduce the duration of delirium. Future studies should conduct a prospective evaluation of remifentanil in similar nonoperative patients in accordance with established protocols.

## Supplementary Information

Supplementary Material 1.

Supplementary Material 2.

## Data Availability

No datasets were generated or analysed during the current study.

## Abbreviations

ICU: Intensive care unit
DFDs: Delirium-free days
LOS: ICU length of stay
APACHE II: Acute Physiology and Chronic Health Evaluation II
SOFA: Sequential Organ Failure Assessment
CRRT: Continuous renal replacement therapy
RASS: Richmond Agitation–Sedation Scale
CPOT: Critical Care Pain Observation Tool
ICDSC: Intensive Care Delirium Screening Checklist
DFDs: Delirium-free days
VFDs: Ventilator-free days
IQRs: Interquartile ranges
PS: Performed propensity score
SMDs: Standardized mean differences
IPTW: Inverse probability of treatment weighting
