# Supplementary material for: Remifentanil use in critically Ill patients requiring mechanical ventilation is associated with increased delirium-free days: a retrospective study
Source: Int J Emerg Med. 2025 Mar 19;18:58. doi: 10.1186/s12245-025-00846-y (PMC11921630; doi:10.1186/s12245-025-00846-y)
Supplement: Supplementary file 1 — Supplementary Material 1. [file 12245_2025_846_MOESM1_ESM.docx]

Appendix 1. STROBE Statement—checklist of items that should be included in reports of observational studies

|  | Item No. | Recommendation | Page  No. | Relevant text from manuscript |
| --- | --- | --- | --- | --- |
| Title and abstract | 1 | 1. Indicate the study’s design with a commonly used term in the title or the abstract | 2 | Abstract |
|  |  | 1. Provide in the abstract an informative and balanced summary of what was done and what was found | 2 | Abstract |
| Introduction | | | |  |
| Background/rationale | 2 | Explain the scientific background and rationale for the investigation being reported | 3 | Introduction |
| Objectives | 3 | State specific objectives, including any prespecified hypotheses | 3 | Introduction |
| Methods | | | |  |
| Study design | 4 | Present key elements of study design early in the paper | 4 | Methods  -  Study design |
| Setting | 5 | Describe the setting, locations, and relevant dates, including periods of recruitment, exposure, follow-up, and data collection | 4 | Methods  -  Study Design and Setting |
| Participants | 6 | (a) Cohort study—Give the eligibility criteria, and the sources and methods of selection of participants. Describe methods of follow-up  Case-control study—Give the eligibility criteria, and the sources and methods of case ascertainment and control selection. Give the rationale for the choice of cases and controls  Cross-sectional study—Give the eligibility criteria, and the sources and methods of selection of participants | 4 | Methods  Patient Selection |
| Variables | 7 | Clearly define all outcomes, exposures, predictors, potential confounders, and effect modifiers. Give diagnostic criteria, if applicable | 4 | Methods  Data Collection |
| Data sources/ measurement | 8* | For each variable of interest, give sources of data and details of methods of assessment (measurement). Describe comparability of assessment methods if there is more than one group | 5 | Methods  Data Collection |
| Bias | 9 | Describe any efforts to address potential sources of bias | 5-6 | Methods  Statistical analysis |
| Study size | 10 | Explain how the study size was arrived at | 5-6 | Methods  Statistical analysis |

Continued on next page

| Quantitative variables | 11 | Explain how quantitative variables were handled in the analyses. If applicable, describe which groupings were chosen and why |  | Methods  Statistical analysis |
| --- | --- | --- | --- | --- |
| Statistical methods | 12 | 1. Describe all statistical methods, including those used to control for confounding | 5-6 | Methods  Statistical analysis |
| Results | | | | |
| Participants | 13* | 1. Report numbers of individuals at each stage of study—eg numbers potentially eligible, examined for eligibility, confirmed eligible, included in the study, completing follow-up, and analysed | 6 | Results  Patient Characteristics |
|  |  | 1. Give reasons for non-participation at each stage | 6 | Results  Patient Characteristics |
|  |  | (c) Consider use of a flow diagram | 6 | Results  Patient Characteristics |
| Descriptive data | 14* | 1. Give characteristics of study participants (eg demographic, clinical, social) and information on exposures and potential confounders | 6 | Results Table1 |
|  |  | *Cross-sectional study—*Report numbers of outcome events or summary measures | 6 | Results – Main results ,  Table 1 |
| Main results | 16 | 1. Give unadjusted estimates and, if applicable, confounder-adjusted estimates and their precision (eg, 95% confidence interval). Make clear which confounders were adjusted for and why they were included | 6 | Results –Main results ,  Table 1.2 |

Continued on next page

| Other analyses | 17 | Report other analyses done—eg analyses of subgroups and interactions, and sensitivity analyses |  |  |
| --- | --- | --- | --- | --- |
| Discussion | | | | |
| Key results | 18 | Summarise key results with reference to study objectives | 7 | Discussion – Key results |
| Limitations | 19 | Discuss limitations of the study, taking into account sources of potential bias or imprecision. Discuss both direction and magnitude of any potential bias | 7 | Discussion – Limitations |
| Interpretation | 20 | Give a cautious overall interpretation of results considering objectives, limitations, multiplicity of analyses, results from similar studies, and other relevant evidence | 8 | Discussion – Conclusion |
| Generalisability | 21 | Discuss the generalisability (external validity) of the study results | 7-8 | Discussion |
| Other information | |  | | |
| Funding | 22 | Give the source of funding and the role of the funders for the present study and, if applicable, for the original study on which the present article is based | 8 | Funding |
