# Supplementary material for: Remifentanil use in critically Ill patients requiring mechanical ventilation is associated with increased delirium-free days: a retrospective study
Source: Int J Emerg Med. 2025 Mar 19;18:58. doi: 10.1186/s12245-025-00846-y (PMC11921630; doi:10.1186/s12245-025-00846-y)
Supplement: Supplementary file 2 — Supplementary Material 2. [file 12245_2025_846_MOESM2_ESM.pptx]

## Slide 1
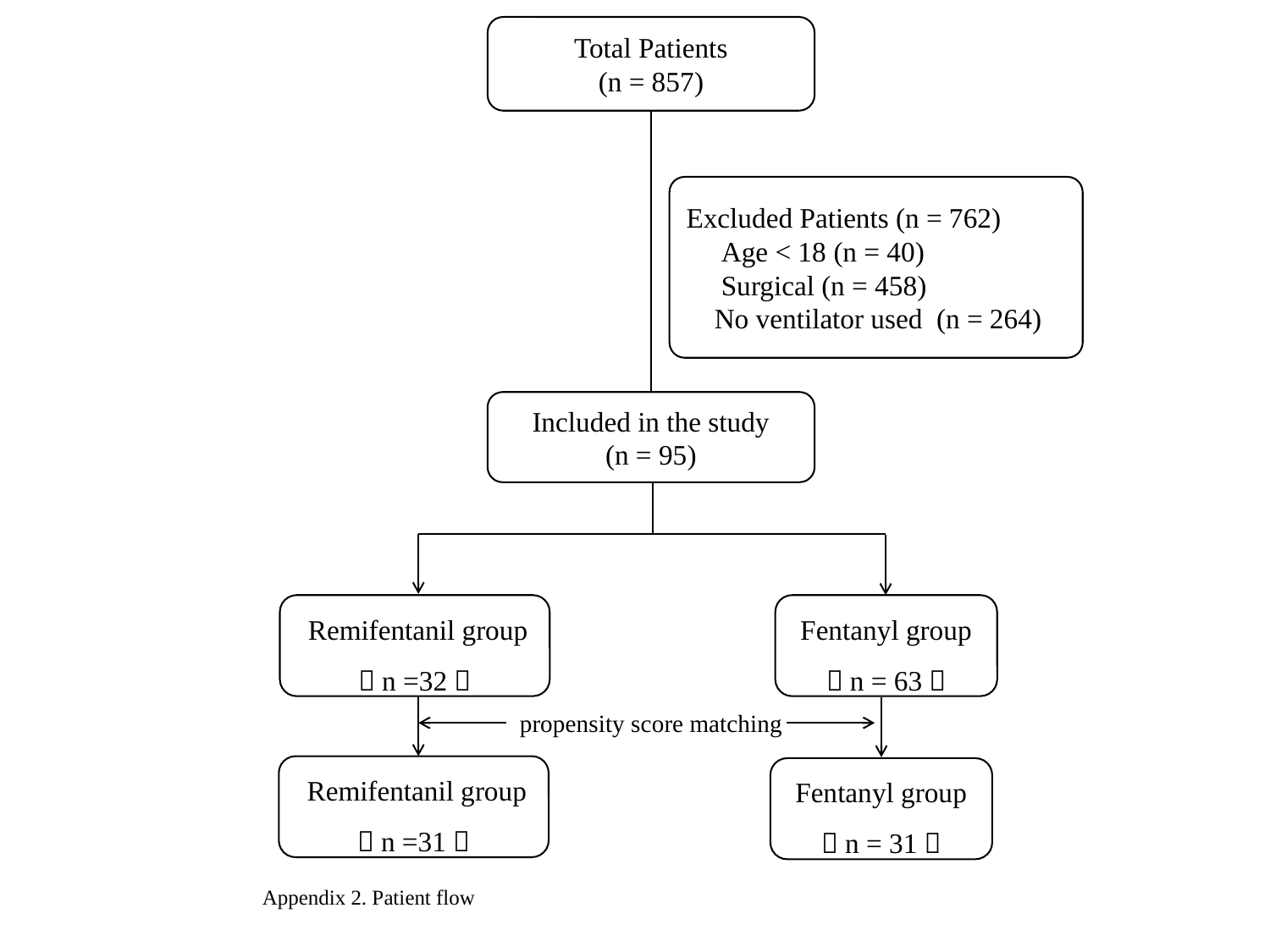

Total Patients
(n = 857)
Excluded Patients (n = 762)
　Age < 18 (n = 40)
　Surgical (n = 458)
 No ventilator used (n = 264)
Included in the study
(n = 95)
 Remifentanil group
（n =32）
Fentanyl group
（n = 63）
propensity score matching
 Remifentanil group
（n =31）
Fentanyl group
（n = 31）
Appendix 2. Patient flow
